# 3D Human Pose Estimation with a Catadioptric Sensor in Unconstrained Environments Using an Annealed Particle Filter

**DOI:** 10.3390/s20236985

**Published:** 2020-12-07

**Authors:** Fakhreddine Ababsa, Hicham Hadj-Abdelkader, Marouane Boui

**Affiliations:** 1 Arts et Métiers Institue of Technology, LISPEN, HESAM University, 75005 Chalon-sur-Saône, France; 2 IBISC Laboratory, University of Evry, 91000 Evry-Courcouronnes, France; Hicham.Hadjabdelkader@univ-evry.fr (H.H.-A.); Marouane.Boui@univ-evry.fr (M.B.)

**Keywords:** human tracking, omnidirectional camera, ego motion, particle filter

## Abstract

The purpose of this paper is to investigate the problem of 3D human tracking in complex environments using a particle filter with images captured by a catadioptric vision system. This issue has been widely studied in the literature on RGB images acquired from conventional perspective cameras, while omnidirectional images have seldom been used and published research works in this field remains limited. In this study, the Riemannian varieties was considered in order to compute the gradient on spherical images and generate a robust descriptor used along with an SVM classifier for human detection. Original likelihood functions associated with the particle filter are proposed, using both geodesic distances and overlapping regions between the silhouette detected in the images and the projected 3D human model. Our approach was experimentally evaluated on real data and showed favorable results compared to machine learning based techniques about the 3D pose accuracy. Thus, the Root Mean Square Error (RMSE) was measured by comparing estimated 3D poses and truth data, resulting in a mean error of 0.065 m when walking action was applied.

## 1. Introduction

Catadioptric sensors are widely used in robotics and computer vision. Their popularity is mainly due to their ability to acquire 360° images with a single shot. They have been used for 3D reconstruction of large environments, robotics, and video surveillance. In addition, 3D human tracking in complex and cluttered environments remains a difficult and challenging problem despite the extensive research work carried out in the literature. In order to get a panoramic view of the environment, several solutions have been proposed using synchronized cameras [1]. However, this kind of system is difficult to implement, especially when the workspace is uncontrolled and cluttered. In this research work, we propose to estimate, through a particle filter, the 3D human pose from images provided by a catadioptric camera. Our main contribution consists in developing robust likelihood functions, which take into account the intrinsic properties of the spherical images. As a result, the particle filter becomes able to propagate the particles in a better manner, which make it more stable and accurate. We provide in detail the architecture of the proposed approach and give more in-depth the experimental results to demonstrate its effectiveness.

The rest of the paper is organized as follows. Section 2 provides the related work. Section 3 describes the proposed particle filter-based 3D tracking approach. Section 4 details the experimental framework undertaken to validate the performance of the proposed algorithm. Finally, some conclusions and future works are drawn in Section 5.

## 2. Related Work

With regard to the state of the art, many research works have been developed for 3D human pose estimation. They can be classified into two main categories: model-based and non-model-based methods. In so-called “model-free” approaches, machine learning techniques [2,3] are often used to estimate a statistical model formalizing the relationship between the human body appearance in images and its 3D posture in the real world. However, this mapping function remains difficult to compute due to the large variability of the articulated human appearance model. With the advent of deep neural networks, significant progress has been made in monocular 3D human pose estimation. Li et al. [4] used a convolutional neural network (CNN) to directly estimate the 3D pose from the image. Tekin et al. [5] use an auto-encoder to learn the pose representation in high dimension and to regress 3D poses from 2D images. Pavlakos et al. [6] proposed a method for fine discretization of the 3D pose by considering the problem as a 3D key point location issue. Then, they used a coarse-to-fine prediction method based on several convolution layers to progressively refine the initial estimates. Other studies have focused on the transition from 2D to 3D. For example, Zhou et al. [7] expressed the optimization problem as the relationship between 2D pose and 3D geometric features, and predicted the 3D pose using an expectation maximization algorithm. Fang et al. [8] have developed a pose grammar to refine the 3D pose using a bidirectional RNN designed to explicitly incorporate a set of knowledge about the human body posture. Chen et al. [9] used the nearest neighbor search method to determine the correspondence between the estimated 2D and 3D poses in a large library of poses. Other works [10,11] adopt adversarial learning to encourage the deep network to acquire plausible human body postures. More recently, Chen et al. [12] present an unsupervised learning approach to generate 3D postures from 2D joints; this approach does not require 3D data but uses geometric self-monitoring as a constraint to learn the 2D-3D lifter. In addition, Habibie et al. [13] propose a deep learning-based architecture for 3D human pose estimation that encodes explicit 2D and 3D features, and uses supervision by a new projection model learned from the predicted 3D poses. Despite the interest in deep convolutional networks, their use in our case would not be appropriate. Indeed, the implementation of deep learning-based solution would have required a large amount of training spherical images with 3D pose annotations. However, to our knowledge, this kind of image dataset does not exist, and the construction of such one requires a lot of time and resources involving a very high cost. In addition, using available perspective image databases during the learning phase will certainly worsen the 3D pose estimation for an omnidirectional input image. Indeed, the intrinsic characteristics of spherical and perspective images are not the same and cannot be correlated in the same way. Furthermore, the “model-based” approaches require an approximation of the real geometry and the movement of the human body. In the literature, different geometric models have been used to represent the human body: articulated model, truncated cylinder, conical, etc. The mechanical and kinematic constraints associated with the human body movements during its displacement are often integrated into the pose estimation process in order to reduce the solution space and thus improve robustness and accuracy. In [14,15] the authors use a “Flexible Mixtures of Parts” [16,17,18] detector to localize in the current image the person’s 2D silhouette. The 3D tracking in the real environment is then determined thanks to a linear regression approach. Moreover, the use of catadioptric cameras for 3D tracking remains very limited despite the many advantages they offer. Many research studies have focused on the development of a specific mathematical model of creating an omnidirectional image while taking into account the particular geometry of the used mirror (hyperbolic, spherical, parabolic) [19]. Other work used omnidirectional cameras to solve classic robotic problems such as visual servoing [20], navigation and motion estimation [21,22]. There is only a few research works using a catadioptric camera for 3D object tracking, like Caron et al. [23] who proposed a sensor composed of four mirrors and a RGB camera. The authors developed a visual servoing approach based on a non-linear pose estimation technique. Their results show a good robustness with regard to illumination changes; however, they only considered simple 3D objects like a box. Tang et al. [24] proposed to model the nonlinear distortions of omnidirectional images using a mixture of Gaussian. The contribution of each part of the human body is weighted according to its detection in the image, making the tracking more robust in case of partial occlusion. However, this approach is applicable only for 2D tracking. In this study, we opted for a tracking scheme based on particle filtering (PF) framework. Indeed, comparing to other approaches, PF is highly accurate and more efficient when dealing with non-linear and non-Gaussian problems. The other advantage to use PF is that the 2D-3D matching between the extracted image features and the 3D model does not need to be explicitly computed, it is implicitly taken into account in the likelihood function. This makes the estimation process more robust against 2D-3D mismatching errors. In addition, FP has the ability to propagate the generated particles uniformly through the solution search space, allowing the method to quickly find the lost target.

## 3. 3D Human Pose Estimation

**Figure 1:**
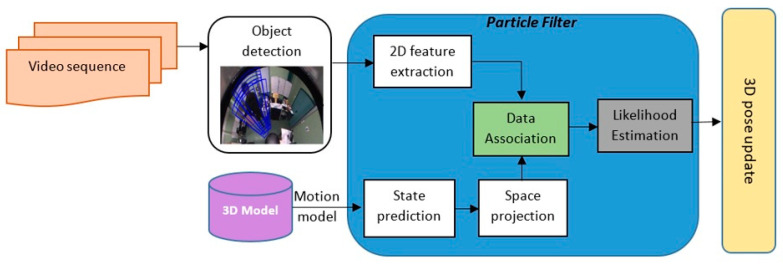
The Overview of the proposed 3D human tracking scheme. HoG (histogram of oriented gradients) features and Support Vector Machine (SVM) classifier are combined to detect the human body in the images. The predicted 3D human model and the extracted 2D features are associated and fed into the Likelihood estimator providing the 3D pose update for the particle filter.

Our approach takes into account the intrinsic geometric properties of the catadioptic sensor as well as omnidirectional images in all steps of pre-processing and pose estimation process (Figure 1). In the detection step, the regions of interest (ROI) corresponding to the human silhouette are extracted and used to initialize the 3D tracking process.

We used HoG (histogram of oriented gradients) descriptors to extract human feature because they effectively describe the local distribution of the human body and they are invariant to illumination changes and small movement in the images. Moreover, linear Support Vector Machines (SVM) trained on HOG features demonstrated an excellent performance for human detection [25]. Thus, the HOG descriptors have been adapted to omnidirectional images before being combining with an SVM classifier. For that, the image gradient is computed in the Riemannian space [2]. The obtained results clearly demonstrate the effectiveness of the catadioptric-adapted gradient comparing to the conventional methods directly computed in the pixel space. Once the tracking initialized, the particle filter allows the generation of several hypotheses of 3D human posture thanks to its particle propagation process around the current pose. Each generated particle corresponds to a probable posture of the 3D human body model in the current image; it takes into account the mechanical and kinematic constraints of the movement due to the articulated aspect of the human body. In order to take into account the distortion caused by the catadioptric sensor, the weight assigned to each particle is computed according to several likelihood functions. The calculation of these functions is given in the following subsections.

### 3.1. The 3D Human Model

**Figure 2:**
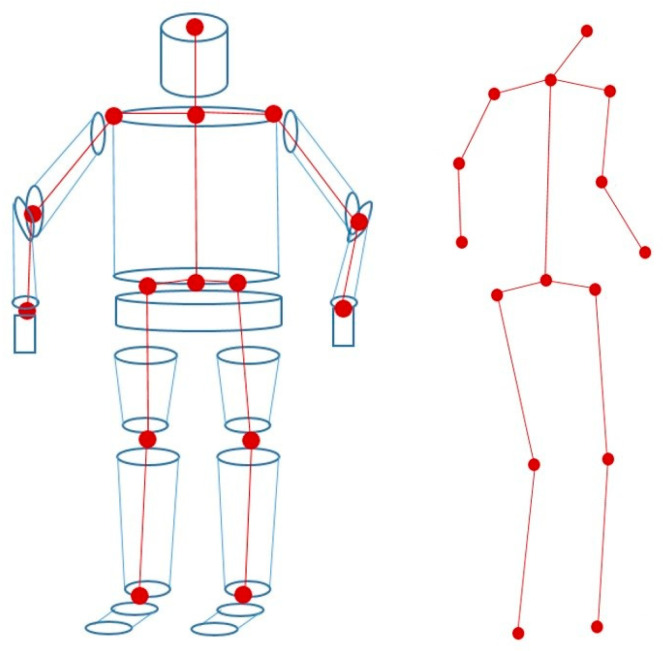
3D Human body model. Head and trunk are modeled by cylinders whereas the upper and lower limbs by truncated cones. 34 degrees of freedom are considered to represent the 3D posture with vertex and joints.

In state-of-the-art research, the human body is often represented by an articulated 3D model whose number of degrees of freedom (DOF) differs according to the application, for example it is equal to 82 in [26] 14 in [27] and 32 in [28]. The number of DOF model directly impacts the behavior of the 3D tracking algorithm, since it corresponds to the vector size of parameters to be estimated. A high number of DOF would increase the estimation time but would allow us to model complex human postures. Recently, more flexible and parameterizable 3D human models have been developed, such as SMPL [29], which allows the representation of different body shapes that deform naturally with the pose, like a real human body. However, this kind of model needs to be trained on thousands of aligned scans of different people in different poses. Their use in our case is not appropriate, as we want to develop a low-cost real-time tracking solution. Thus, we opted for cylinders to model the head and trunk of the human body, and truncated cones for the upper and lower limbs (Figure 2). This representation has the advantage of being simple to handle (few parameters to define a cylinder/cone) [30,31] and easy to project into images. Our model has 34 degrees of freedom, composed of 11 parts: pelvis, torso, head, head, arms, forearms, legs and thighs. The model shape is represented by the length and width of the upper/lower limbs and trunk, while the 3D posture is defined through 30 parameters that give the position and orientation of the pelvis as well as the angles at the joints between the different body parts. In the end, all these parameters were grouped into a single vector x=[x(1),x(2),…,x(29),x(30)] that defines a complete 3D model of the human body.

**Figure 3:**
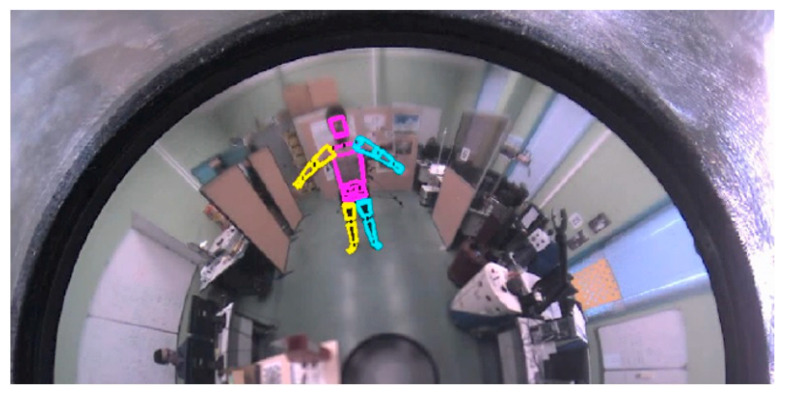
3D human body model projected on the omnidirectional image. The geometrical model of the Catadioptic sensor has been taken into account in the projection process.

In addition, used the unified model to take into account the geometry of the catadioptic sensor when projecting the 3D model into the current image. Thus, the projection of a straight-line segment gives conics on the image plane (Figure 3).

### 3.2. The Filtering

Filtering consists in estimating the current state $x_{t}$ taking into account all past measurements $y_{1:t}\equiv \left\{y_{1},\ldots ,y_{t}\right\}$ [32]. From a mathematical point of view, this results in estimating the posterior distribution of the current state $p\left(x_{t}|y_{1:t}\right)$. In our case, the state vector includes all the parameters describing the 3D posture of the human body as explained in the previous section, and the measurements that feed the filter at each iteration correspond to visual primitives extracted from the current image. The posterior distribution of the current state $p\left(x_{t}|y_{1:t}\right)$ can be recursively computed from the distribution of the previous state $p\left(x_{t-1}|y_{1:t-1}\right)$ in two steps:

- Prediction step:
  (1) $$p\left(x_{t}|y_{1:t-1}\right)=\int p\left(x_{t}|x_{1:t-1}\right)\cdot p\left(x_{t-1}|y_{1:t-1}\right)\cdot dx_{t-1}$$
- Update step
  (2) $$p\left(x_{t}|y_{1:t}\right)\propto p\left(y_{t}|x_{t}\right)\cdot p\left(x_{t}|y_{1:t-1}\right)$$

In Equation (1) the temporal diffusion model $p\left(x_{t}|x_{1:t-1}\right)$ is used to compute the predicted state. In this study, we use the random walk model that gives the best results when setting the standard deviations at 0.1 m for translation and 1.5° for rotation. The filtered solution (posterior distribution) corresponds to the predicted pose weighted by the likelihood function $p\left(y_{t}|x_{t}\right)$, which corresponds to the observation probability conditioned by the estimated pose. It is known that the filtering equations can generally not be solved in closed form, except for linear Gaussian systems where the Kalman Filter (KF) provides the exact solution [24]. A large amount of research has been carried out to generalize the KF solution to non-linear systems. Different numerical methods have been developed such as the EKF (Extended Kalman Filter). In this work, we used the Particle filter framework for its simple implementation and its effectiveness in managing complex and random motion. So, we implemented an annealed particle filter (APF) which is based on Sequential Importance Resampling (SIR) algorithms [33,34] or CONDENSATION algorithm [35]. The APF filter was developed by Deutscher et al. [36] to solve the problem of articulated body motion tracking with a large number of degrees of freedom. The basic principle of the APF is the use of the annealing in an iterative way in order to better estimate the peaks of the probability density. Therefore, at each time, the APF algorithm proceeds in a set of “layers”, from layer M down to layer 1, that update the probability density over the state parameter. A series of weighting functions are employed in which each $w_{m}$ differs only slightly from $w_{m+1}$, where $w_{m}$ is designed to be very broad representing the direction of the search space. The posterior distribution after each layer m+1 of an annealing run is represented by a set of N weighted particles: $S_{t,m+1}={\left\{x_{t,m+1}^{i},\pi_{t,m+1}^{i}\right\}}_{i=1}^{N}.$ For the prediction step at layer m, a Gaussian diffusion model is implemented. Specifically, a “Monte Carlo sampling with replacement” method is used to generate the new hypotheses at layer m from the posterior density at the previous layer m+1 using:

(3) $${\left\{x_{t,m}^{\left(i\right)}\right\}}_{i=1}^{N}\approx \sum_{j=1}^{N}\pi_{t,m+1}^{\left(j\right)}N\left(x_{t,m+1}^{\left(j\right)},\alpha^{M-m}C\right)$$

The sampling covariance matrix C controls the extent of the research space at each layer, where a large covariance matrix allows for a more widespread distribution of the sampled particles. The Parameter α is used to gradually reduce the covariance matrix C in the lower layers in order to guide the particles to the modes of the posterior distribution. In our case, α is set at 0:4. Sampled poses that do not respect the geometric constraints of the articulated model of the human body (limits of the articular angle of the model exceeded or interpenetration of the limbs) are rejected and are not resampled in a layer. New normalized weights are assigned to the remaining particles based on an “annealed” version of the likelihood function:

(4) $$\pi_{t,m+1}^{\left(j\right)}=\frac{p{\left(y_{t}|x_{t,m}^{\left(i\right)}\right)}^{\beta^{m}}}{\sum_{j=1}^{N}p{\left(y_{t}|x_{t,m}^{\left(j\right)}\right)}^{\beta^{m}}}\;i\in 1,\ldots ,N$$

The value of $\beta^{m}$ will determine the annealing rate at each layer. Generally, the parameter $\beta^{m}$ is set so that about half of the particles are propagated to the next layer by Monte-Carlo sampling.

### 3.3. Likelihood Functions

The likelihood of each particle in the posterior distribution measures how well the projection of a given body pose fits the observed image. Therefore, it is important to correctly choose, which image features are to be used to construct the weighting function. Many image features could be used, including appearance models and optical flow constraints. In our case, we use edge and silhouette features for their simplicity (easy and efficient to extract) and their degree of invariance to imaging conditions, namely with omnidirectional images.

#### 3.3.1. Edge-Based Likelihood Function

The image gradient is first used to detect the edges in the omnidirectional images. Then, we propose to use geodesic metrics to process spherical images and measure the distance between a pixel and the edge. For that, a gradient-mapping on the Riemannian manifold [30,31] is considered. Let S be a parametric surface on ${\mathbb{R}}^{3}$ with an induced Riemannian metric $g^{ij}$ that encodes the geometrical properties of the manifold. A point on the unit sphere can be represented according to Cartesian or polar coordinates by (x,y,z)=(sinθsinϕ,sinθcosϕ,cosθ). The Riemannian inverse metric is then given by:

(5) $$g^{ij}=\gamma \left(\begin{array}{ll} -x^{2}\left(\xi -1\right)+\xi +1 & xy\left(\xi -1\right)\\ xy\left(\xi -1\right) & -y^{2}\left(\xi -1\right)+\xi +1 \end{array}\right)$$

where

(6) $$\gamma =\frac{{\left(x^{2}+y^{2}+{\left(1+\xi \right)}^{2}\right)}^{2}}{\left(1+\xi \right){\left(\xi +\xi^{2}+\sqrt{1-\left(x^{2}+y^{2}\right)\left(\xi^{2}-1\right)+2\xi +\xi^{2}}\right)}^{2}}$$

and ξ is a projection parameter which takes into account the shape of the mirror. When ξ=0 we are back to the pinhole model.

This Riemannian metric is then used as a weighting function applied to the classical gradient computed on the omnidirectional image:

(7) $$\nabla f=g^{ij}\frac{\partial f}{\partial x_{i}}$$

and on the spherical images:

(8) $$\nabla_{S^{2}}I_{S}\left(\theta ,\phi \right)=\frac{\partial I_{S}\left(\theta ,\phi \right)}{\partial \theta }e_{\theta }+\frac{1}{sin\theta }\frac{\partial I_{S}\left(\theta ,\phi \right)}{\partial \phi }e_{\phi }$$

where (θ,ϕ) represent, respectively, the longitude and colatitude angles; and $\left(e_{\theta },e_{\phi }\right)$ are the unit vectors.

For each pose hypothesis (defined by a particle of the APF filter), the 3D human model is projected into the generated gradient image. Then the distance between the projected model and the contour is determined. In omnidirectional images, the distance between two neighboring pixels differs according to the image region under consideration and therefore using the Cartesian distance is not suitable. We have therefore opted for the geodesic distance in order to build the distance map. The geodesic distance between two points in a spherical image, $x_{1}=\left(\theta_{1},\phi_{1}\right)$ and $x_{2}=\left(\theta_{2},\phi_{2}\right)$, is given by:

(9) $$d_{S^{2}}\left(x_{1},x_{2}\right)=arcos\left(\left[\begin{array}{c} cos\phi_{1}sin\theta_{1}\\ sin\phi_{1}cos\theta_{1}\\ cos\theta_{1} \end{array}\right]\cdot \left[\begin{array}{c} cos\phi_{2}sin\theta_{2}\\ sin\phi_{2}cos\theta_{2}\\ cos\theta_{2} \end{array}\right]\right)$$

**Figure 4:**
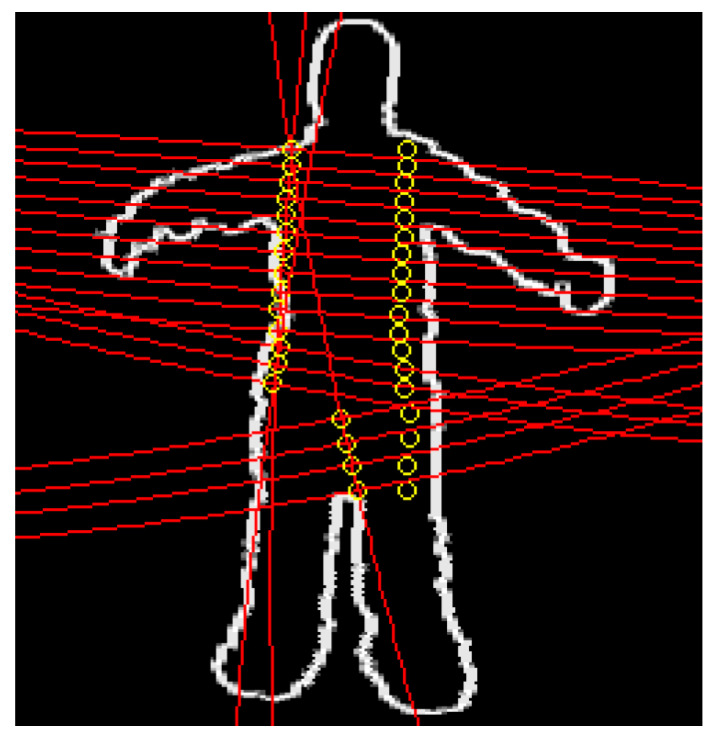
Geodesic distances in the spherical image. An example of sampled points from a part of the 3D model projected in the image. The dots represented by yellow circles correspond to the sampled points of a part of the 3D model projected in the image.

An edge distance map $M_{t}^{e}$ is then constructed for each image. The likelihood is estimated by projecting the complete model into the edge map and computing the mean squared error:

(10) $$p^{e}\left(y_{t}|x_{t}\right)\propto \frac{1}{\xi_{x_{t}}^{e}}\sum {\left(1-M_{t}^{e}\left(\xi_{x_{t}}^{e}\left(j\right)\right)\right)}^{2}$$

where $\xi_{x_{t}}^{e}\left(j\right)$ represents the coordinates of the image points corresponding to the projected 3D model points in the image along all the body parts, using the estimated 3D pose $x_{t}$. In order to improve the computing speed, we calculate the geodesic distance according to given direction. Thus, the large circle that passes through the ends of each 3D model cylinder is determined. Then, several circles belonging to the perpendicular planes on this large circle are generated in order to sample the projected 3D model. The points of intersection between these circles and the cylinder contour correspond to the sample points of the projected 3D model (Figure 4). This reduces the number of pixels whose distance from the edge must be calculated. Indeed, unlike the case of perspective images, the complexity of the Distance Map calculation is very high when spherical images are considered.

#### 3.3.2. Silhouette-Based Likelihood Function

The scene background is estimated using a Gaussian mixture model, then subtracted at each time to generate the binary foreground silhouette map $M_{t}^{s}$. The silhouette likelihood function is then estimated by the equation:

(11) $$p^{s}\left(y_{t}|x_{t}\right)\propto \frac{1}{\xi_{x_{t}}^{s}}\sum {\left(1-M_{t}^{s}\left(\xi_{x_{t}}^{s}\left(j\right)\right)\right)}^{2}$$

However, this function constrains the body to lie inside the image silhouette. In order to correct this defect, we define a new silhouette likelihood that penalizes non-overlapping regions. Let $M_{t}^{p}$ represents the binary Body model silhouette map obtained by projecting the 3D model to the spherical image. Three regions can then be defined to estimate the likelihood of the overlap region between the two silhouettes $M_{t}^{s}$ and $M_{t}^{p}$: the overlap region $R_{t}^{1}$ corresponding to the intersection between $M_{t}^{s}$ and $M_{t}^{p}$, $R_{t}^{2}$ and $R_{t}^{3}$ regions corresponding to the difference between the $M_{t}^{s}$ and $R_{t}^{1}$ on one side, and $M_{t}^{p}$ and $R_{t}^{1}$ on the other side. The size of each region can be computed by summing all the image pixels as follows:

(12) $$R_{t}^{1}=\sum_{i}M_{t}^{p}\left(i\right)\cdot M_{t}^{s}\left(i\right)$$

(13) $$R_{t}^{2}=\sum_{i}M_{t}^{s}\left(i\right)\cdot \left(1-M_{t}^{p}\left(i\right)\right)$$

(14) $$R_{t}^{3}=\sum_{i}M_{t}^{p}\left(i\right)\cdot \left(1-M_{t}^{s}\left(i\right)\right)$$

Thus, the dual likelihood function is defined as a linear combination of these regions:

(15) $$p^{sd}\left(y_{t}|x_{t}\right)\propto \frac{1}{2}\left(\frac{R_{t}^{2}}{R_{t}^{1}+R_{t}^{3}}+\frac{R_{t}^{3}}{R_{t}^{1}+R_{t}^{3}}\right)$$

Finally, we use the multiple probability formulation to combine the different likelihood functions:

(16) $$p\left(y_{t}|x_{t}\right)=\frac{1}{\left|L\right|}\sum_{l\in L}-Log\left(p^{l}\left(y_{t}|x_{t}\right)\right)$$

where $y_{t}$ is the image observations obtained at time t and L∈{e, s, sd} is the set of the proposed likelihood functions.

## 4. Experimental Results

In this section, we detail the experiments we have carried out under real conditions to study the behavior of our 3D tracking algorithm and to evaluate its performance. We used the SmartTrack “capture motion” system [37] to generate the ground-truth of the 3D body poses. We first detail the experimental protocol put in place, as well as the construction of our test database, and then we present the used evaluation criteria and discuss the obtained results.

### 4.1. Acquisition System Setup

**Figure 5:**
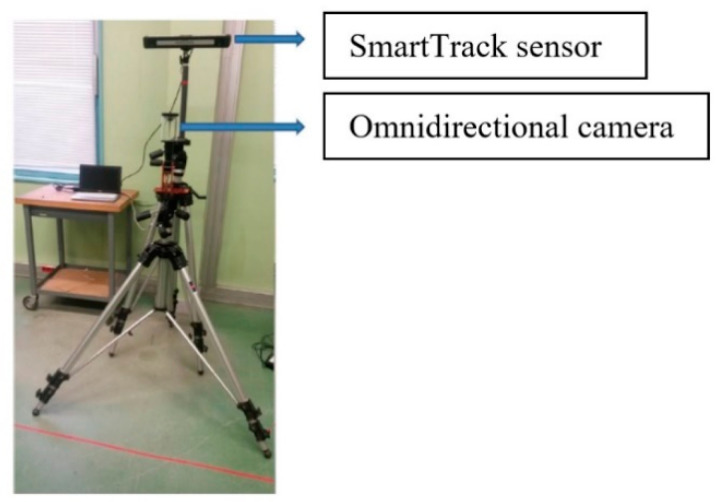
Data acquisition setup. The SmartTrack device and the Omnidirectional camera are mounted on a tripod. A calibration process was carried out to determine the rigid transformation between the two systems.

The used acquisition system is composed of the SmartTrack device and an omnidirectional camera realized by combining a hyperbolic mirror with a perspective camera, as shown in Figure 5.

The SmartTrack is an integrated tracking system. This means, inside the small housing are integrated not only two tracking cameras but also the Controller, which performs all calculations and generates the data output stream. It is composed of two infrared (IR) cameras with a field of view of approximately 100 degrees in horizontal and 84 degrees in vertical. The IR cameras allow the tracking of targets within reflective surface. Indeed, these markers reflect the incoming IR radiation into the direction of the incoming light. More precise: the IR radiation is reflected into a narrow range of angles around the (opposite) direction of the incoming light. Passive markers are mostly spheres covered with retro reflecting foils. However, they can also be stickers made from retro reflecting material. In our experiment, we placed the passive markers on the person’s pelvis and head to record their 3D position and orientation in real time. We used a WIA (Windows Image Acquisition) server to synchronize the data provided by the SmartTrack device with the images acquired from the omnidirectional camera.

### 4.2. Database Construction

**Figure 6:**
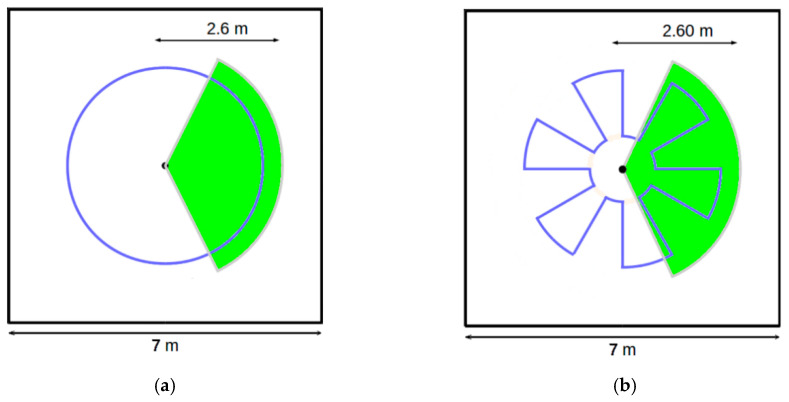
Path Movement for the three sequences. The blue lines correspond to the path followed by the person during his movement around the sensor. The green areas represent the regions where the person is tracked by the SmartTrack system. (**a**) Sequence 1 and 2, (**b**) Sequence 3.

Thanks to the acquisition system, we built a database composed of four sequences. The first one represents a person moving slowly around the sensor (Figure 6a). In sequence 2, the person follows the same trajectory as in sequence 1 with an oscillating movement of his arms. In the third sequence, a movement around the sensor with a forward/backward motion has been performed (Figure 6b).

The fourth sequence presents a more complex scenario where the person rotates around himself and climbs stairs. This sequence allows us to evaluate the robustness of the algorithm against the self-occlusion problem. The video sequences were captured at frame rate of 25 images per second. The characteristics of the collected dataset are summarized in Table 1.

### 4.3. Performance Criteria

We use two evaluation metrics based on the mean square error (MSE) [38,39] to compare the estimated body poses given by our algorithm and the truth data. The first one computes the average Euclidean distance between the markers placed on the joints and extremities of the limbs and the estimated poses. This distance is given by:

(17) $$D_{3}\left(x,\tilde{x},\right)=\frac{1}{N}\sum_{i=1}^{N}{\left|m_{i}\left(x\right)-m_{i}\left(\tilde{x}\right)\right|}^{2}$$

where $m_{i}\left(x\right)\in {ℛ}^{3}$ are the locations of the markers corresponding to the 3D ground truth, and $m_{i}\left(\tilde{x}\right)$ represent the 3D joint positions induced by the estimated pose $\tilde{x}$.

The second criterion is a pixellic distance measured in the images. To do this, we manually annotated the videos in the dataset with extra information representing the ground-truth of the body posture in the image sequence. Thus, for each frame of each video, we annotated the positions of 11 ends of the human silhouette. For the evaluation, we project the human body model into the images and then compute the 2D distance between the projected ends and the annotated dataset, as follows:

(18) $$D_{2}\left(x,\tilde{x}\right)=\frac{1}{N}\sum_{i=1}^{N}{\left|p_{i}\left(x\right)-d_{i}\left(\tilde{x}\right)\right|}^{2}$$

where $p_{i}\left(x\right)$ is the 2D points annotated in the reference image of the database, $d_{i}\left(\tilde{x}\right)\in {ℛ}^{2}$ is the projection in the image of the 3D coordinates of the target i knowing the predicted pose $\tilde{x}.$

### 4.4. Evaluation of the APF Parameters

**Figure 7:**
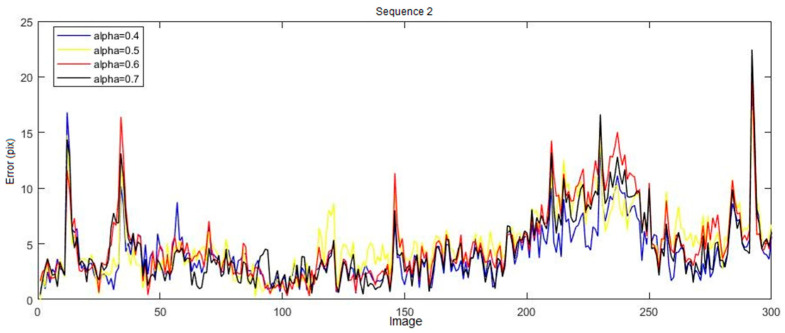
Influence of the parameter α—sequence n° 2. The results suggest that a good choice for the alpha parameter can improve the performance of the annealed particle filter (APF) and consequently increase the accuracy of 3D tracking.

Given the stochastic nature of our 3D tracking approach, the results obtained when performing the same experiment with the same APF configuration parameters often support different results. Thus, to obtain consistent measurements and repeatability of the performance, each experiment is run 10 times for each sequence. We calculate the average of the errors (3D or 2D) obtained at each moment on all the estimated positions. First, we evaluated the effect of the resampling parameter α used in the APF to limit the spread of particles from layer M to layer M−1. It can be seen, as shown in Figure 7, that this parameter has an important influence on the obtained results, especially when the number of particles is low.

Thus, we varied the value of the parameter α from *0.2* to *0.7* and compute the average data of the 2D error for all sequences. The obtained results are summarized in Table 2. We can see that that the value α=0.4 allows us to obtain the best performances for all sequences. Indeed, this value allows the constraint of the propagation space from one layer to the next when the human movements are significant. This is the case with the arms in sequence 2 where the system no longer allows us to track the joints that have undergone a great movement. For example, the obtained 2D error (in pixels) when α=0.4 is about 4.15±0.73 pixels for sequence 2. α=0.6 gives the poorest results with an error of 5.06±1.16 pixels. Therefore, appropriate choice of the parameter α can improve the tracking performance by 22%.

### 4.5. Comparing of Likelihood Functions

**Figure 8:**
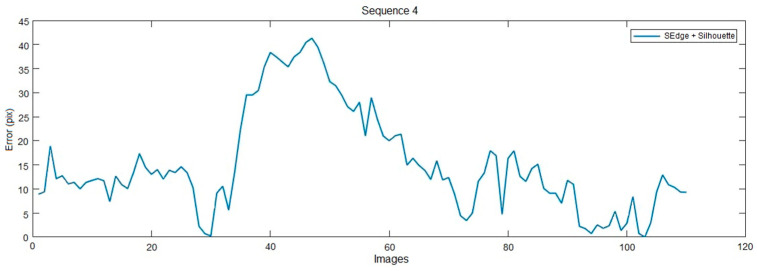
Obtained results on sequence 4 using the combined likelihood function (DS+GG). Average 2D distance between the projected 3D model and the annotated data. This error increases significantly when the tracking of the upper limbs is lost due to self-occlusion, this is the case between frame 40 and 50.

In this section, the effect of the likelihood function on the performance of the proposed 3D tracking algorithm is studied. Thus, four likelihood functions are considered: Spherical Gradient with Geodetic Distance (GG) (defined by Equation (10)), Omnidirectional Gradient (OG), Dual Silhouette (DS) (defined by Equation (11)), and a combination of DS and GG likelihood functions (given by Equation (15)). As a reminder, the likelihood function (OG) uses the classical gradient function (Equation (7)) weighted by the Riemannian metric and calculated on the omnidirectional image. The results obtained when we apply our approach to sequence 1 and 2 demonstrate that GG likelihood function performs better than OG function. It improves the accuracy by 11% compared to the OG function. This demonstrates that handling omnidirectional images in spherical space and using the geodesic distance increases the pose estimation quality. The second result that is clearly seen is that the combination of the likelihood functions DS and GG always gives the best results. Figure 8 shows the obtained results for sequence 4 using DS + GG likelihood function; we found an average error of 15 pixels per image. This is because of the complexity of sequence 4, which presents many self-occlusions of the upper and lower limbs.

Table 3 summarizes, for each sequence, the average pixel error obtained for the proposed likelihood functions (computed using Equation (17)). It can be seen that this error is in the range of 4.15 to 7.95 pixels for sequences 1, 2 and 3, whereas it reaches 22 pixels for sequence 4. This can be explained by the fact that sequence 4 has self-occlusion of the upper limbs. Thus, when the person rotates on itself, and the arms remain stuck along the body, then neither the contour nor the silhouette can provide enough information to detect the person’s rotation.

**Figure 9:**
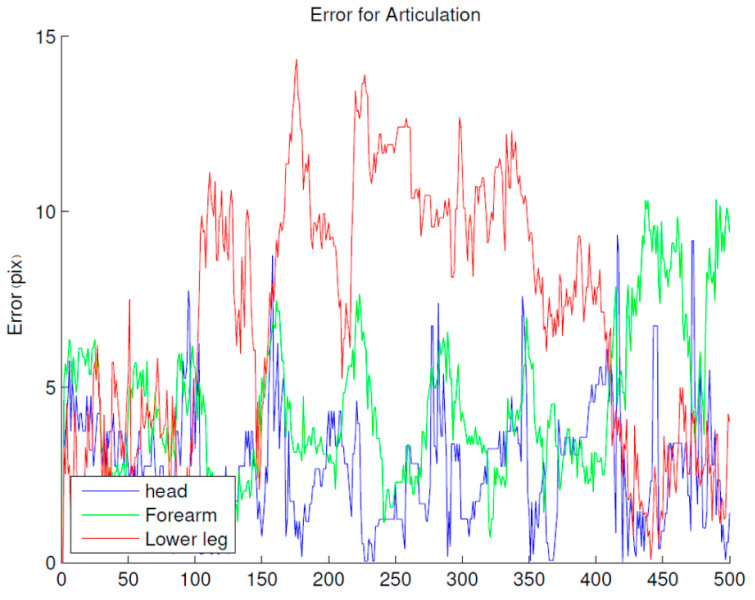
Tracking errors of the body extremities. Placing the omnidirectional camera at a height of 1.5 m allows the person’s head to be visible in all images, which facilitates its tracking and explains the good obtained accuracy.

Figure 9 shows the tracking results of the body extremities: head, hands and feet. We note that the head is the part of the body that is best tracked, while the feet are less well tracked. Indeed, the position of the feet in the omnidirectional images are close to the center, which reduces their size and makes their detection more difficult.

**Figure 10:**
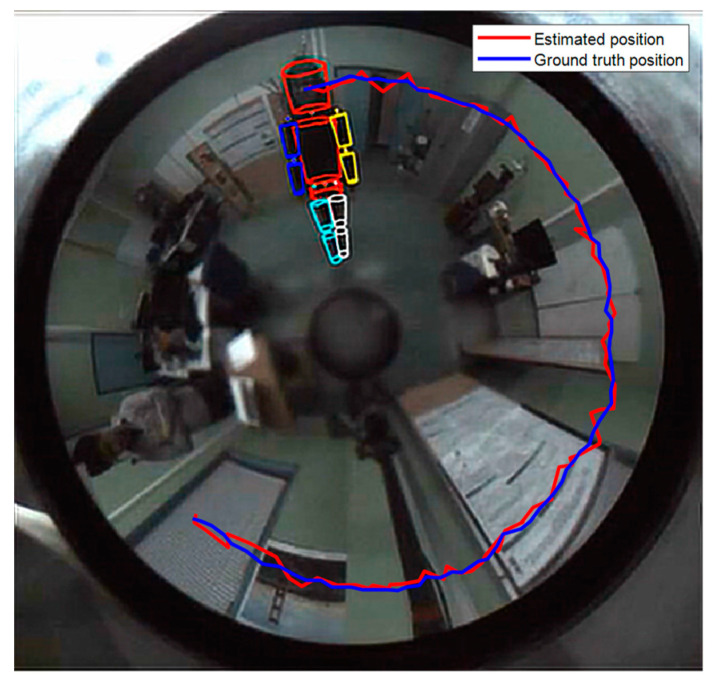
Head tracking results. The average location error between the estimated 3D pose of the head and the ground-truth data is about 20 mm.

Figure 10 illustrates an example of head tracking compared to ground truth. The blue and red trajectories on the image correspond to the history of the estimated and real head positions, projected into the current image. We can see that the head displacement estimated by our tracking algorithm corresponds to the real trajectory recorded by the SmartTarck system. This demonstrates the accuracy of our approach and its effectiveness when processing real data.

### 4.6. Evaluation of the Computation Time

The computation time is directly proportional to the number of particles as well as to the number of layers of the APF filter. It also depends on likelihood functions. Table 4 summarizes the computation times obtained for the slowest case when a combination of two likelihood functions (gradient with geodesic distance and dual silhouette) is used with 100 particles for a single layer (the computation time of propagation likelihood function will be multiplied by the number of layers). The computation time to perform the 3D tracking on one frame of 800 × 600 pixels is about 0.79 s when using a 3 GHz Intel Core-i7 with Matlab implementation. We note that the time required for image pre-processing (calculation of the gradient and geodetic distance) represents about 57% of the total computing time. This high time is mainly due to the multiple omnidirectional projections towards the spherical space. In our case, we limit the calculations to a restricted image space thanks to the HOG detection window. In addition, the time required to estimate the likelihood functions represents 37% of the overall calculation time, while the time required to propagate the particles of the APF filter and subtract the background is relatively small; it represents only 1% of the total time.

### 4.7. Comparison with Other Works

For completeness, we present a qualitative analysis that compare our results against other 3D human pose estimation methods. This is just meant to be an indicative result, as the considered methods are evaluated differently. Indeed, public omnidirectional image datasets are unfortunately not available, which did not allow us to carry out a quantitative comparison with state-of-the-art techniques. We evaluate the accuracy of 3D human pose estimation in terms of average Euclidean distance between the predicted and ground-truth 3D joint positions and Head. We compare the results obtained from the “walking” action in our investigation with recent state-of-the-art approaches which are tested in the walking action of popular public datasets like Human3.6M and HumanEva-I. The walking action in our database corresponds to one person’s movement towards the camera, with a coherent swing of the left (right) arm and the right (left) leg with each other in space, which is quite similar with the walking action of Human3.6M and HumanEva-I databases. The reported results are presented in Table 5. We can see that the performance of our approach is similar to state-of-the-art methods, validating the effectiveness of our tracking scheme. Nevertheless, it would be interesting to generalize this result by testing the robustness of our approach under more challenging conditions with complex human actions.

## 5. Conclusions

This paper presents a new approach for human pose estimation by using a catadioptric vision system within the context of Bayesian filtering. We developed original likelihood functions in Riemannian/spherical space to take into account the geometrical properties of the omnidirectional images. The spherical image derivatives were then used to adapt the gradient computation to this space, and the geodesic distance was considered when generating the distance map. Numerous experiments were carried out with real image sequences to evaluate the performance of the proposed approach. We used the MSE criteria to measure the quality of the estimated 3D pose in comparison to the ground truth data. The results show that the performance is further improved when using the combined Silhouette/Edge likelihood function. Indeed, our algorithm converges in less than 1 s in most cases, while the 3D pose estimation error generally remains below 10 cm. However, we have observed that the AFP filter sometimes has some limitations, in particular, when the body extremities are partially occluded or when the person is more than 5 m away from the sensor. As future work, we plan first to explore the use of additional information provided by other sensors, like Kinect and IMU (Inertial Measurement Unit), to improve the estimation accuracy, and second to use deep learning approaches such as those that have been demonstrated to produce remarkable results for classical 3D object localization.

## Figures and Tables

**Table 1:** Specification of the collected video dataset.

Characteristics	Sequence 1	Sequence 2	Sequence 3	Sequence 4
Number of frames	600	682	768	432
Duration (second)	49	56	63	35
Kind of movement	Circular	Circular with arms	Forward/Backward	Walk/occlusion

**Table 2:** Average 2D error according to the parameter α.

Sequences	α=0.2	α=0.4	α=0.6
Sequence 1	6.48±1.02	6.04±0.94	6.32±1.26
Sequence 2	4.51±0.82	4.15±0.73	5.06±1.16
Sequence 3	7.36±1.42	6.54±1.03	7.03±1.21
Sequence 4	7.94±1.73	7.09±1.14	7.52±1.42

**Table 3:** The Mean localization error in the image (pixels) of different sequences in the database.

Likelihood Functions	Sequence 1	Sequence 2	Sequence 3	Sequence 4
DS	6.86 ± 0.70	7.15 ± 0.65	7.95 ± 0.76	20.15 ± 1.51
OG	6.37 ± 0.60	8.15 ± 0.72	7.01 ± 0.73	22.00 ± 1.86
GG	4.40 ± 0.45	5.70 ± 0.53	7.20 ± 0.62	18.40 ± 1.63
DS + GG	4.15 ± 0.63	5.30 ± 0.58	6.72 ± 0.61	15.20 ± 1.26

**Table 4:** Computation times for our 3D tracking algorithm (100 particles, m = 1).

Image Size	800 × 600	1028 × 738
Subtracting the background	0.0067 s (1%)	0.0073 s (1%)
Gradient + geodesic distance computation	0.39 s (59%)	0.46 s (58%)
Propagation	0.032 s (5%)	0.043 s (5%)
Likelihood functions computation (dual silhouette)	0.23 s (35%)	0.28 s (36%)
Total	0.66 s	0.79 s

**Table 5:** 3D errors (mm) of 3D human pose estimation methods in the walking actions.

Methods	Evaluation Datasets	Error
Pavlakos et al. [6]	Human3.6M	59.1
Fang et al. [8]	Human3.6M	47.5
Chen et al. [9]	Human3.6M	55.7
Habibie et al. [13]	Human3.6M	54.3
Wang et al. [40]	HumanEva-I	71.1
Makris et al. [41]	Berkeley MHAD	80.0
Our approach	Our own dataset	64.7
